# Safety and Efficacy of Risankizumab and Infliximab in the Treatment of Plaque Psoriasis: Results From a Direct and Indirect Meta-Analysis

**DOI:** 10.7759/cureus.15963

**Published:** 2021-06-27

**Authors:** Mohammad Almohideb

**Affiliations:** 1 Dermatology, College of Medicine, King Saud Bin Abdulaziz University for Health Sciences, Riyadh, SAU

**Keywords:** risankizumab, infliximab, psoriasis, meta-analysis, direct

## Abstract

The objective of our study was to compare a potent drug of the anti-TNF class family, infliximab, with a potent drug of the IL-inhibitors family, risankizumab, in terms of efficacy and safety endpoints. Online databases were searched for relevant placebo-controlled, randomized trials. The following efficacy outcomes were included: PASI-75, PASI-90, and sPGA, as well as the incidence of any adverse events and serious adverse events. The risk ratios (RR) with the respective 95% confidence intervals (CIs) of different psoriasis scores were pooled in a meta-analysis model, using the Mantel-Haenszel method.

The combined risk ratios (RR) showed that infliximab and risankizumab are effective in increasing the number of patients with more than 75% improvement in the PASI (RR= 26.68, 95% CI [14.98, 47.51] p<0.001) and (RR= 10.17, 95% CI [7.24, 14.30] p<0.001), respectively. Test for subgroup differences showed that risankizumab is more effective. Regarding PASI-90 outcome, risankizumab and infliximab are more effective than placebo (RR= 26.22, 95% CI [14.20, 48.41], p<0.001), and (RR= 15.18, 95% CI [8.72, 26.45], p<0.001) respectively. The results showed that risankizumab does not cause significant serious adverse events (RR = 0.59, 95% CI [0.31, 1.13], p=0.12) while, on the other hand, infliximab causes significant serious adverse events (RR = 2.30, 95% CI [1.08, 4.88], p=0.03). The test of subgroup difference showed that risankizumab is safer (p<0.001). Analysis of the incidence of any adverse events showed that risankizumab is safer as well (p=0.007). Infection rates were similar among both drugs (p=0.05). In conclusion, risankizumab is preferred for the treatment of psoriasis than infliximab, and is significantly more effective and safe.

## Introduction and background

Psoriasis is a skin disorder characterized by chronic inflammation, epidermal hyper-proliferation, and vascular changes [1]. The prevalence of psoriasis ranges from 0.5% to 11.5% worldwide [2]. This condition causes itching of variable severity as well as many psychological disturbances including depression, feelings of inferiority and anxiety [3].

The pathogenesis of the disease is still not clearly known; however, it is confirmed that T-lymphocytes play a major role. Psoriatic T-cells are basically activated memory lymphocytes which secrete cytokines that targets the skin and cause psoriatic skin features [4]. Tumor necrosis factor-alpha (TNF-α) is a common cytokine that is thought to increase cellular expression of vascular endothelial growth factors from the skin, leading to angiogenesis and epidermal proliferation [5].

Interleukin (IL) 23 has been found to play a role in the development of the disease as well [6]. Current treatment approaches aim at inhibiting these two major pathways, through either TNF-α inhibitors or IL-23 antagonists.

Risankizumab is a recent humanized monoclonal antibody from the anti-IL family that has shown great efficacy. It acts by inhibiting IL-23 through irreversible binding to the p19 subunit of the cytokine [7]. A network meta-analysis published recently demonstrated that risankizumab shows the most efficacy and lowest risk compared to other drugs such as brodalumab, secukinumab, ixekizumab, ustekinumab, guselkumab, or tildrakizumab [8]. Another recent study showed that IL-inhibitors improve the Dermatology Life Quality Index in patients with psoriasis [9].

Patients treated with IL-inhibitors reported better scores after 12 weeks of therapy compared to the placebo. Infliximab, from the anti-TNF family, is a murine-human monoclonal antibody that was approved by the US food and drug administration (FDA) in 2006 [10]. It acts through interfering with the binding of the cytokine to its receptor [11].

Infliximab holds the highest efficacy records compared to other drugs of the same family such as etanercept, efalizumab, and alefacept [12]. The drug has shown great success in improving DLQI scores, and the improvement is increased by increasing the dose [13].

Many studies have compared different drugs of both families in the treatment of psoriasis. However, there is no evidence of a comparison between risankizumab and infliximab. This systematic review and meta-analysis aims to perform an indirect comparison on a statistical basis between the most effective drug of the anti-TNF family, infliximab, and the family of IL-inhibitors, risankizumab.

## Review

Materials and Methods

This systematic review and meta-analysis complies with the Preferred Reporting Items for Systematic Reviews and Meta-Analyses (PRISMA) guidelines [14]. All steps were performed in accordance with the Cochrane’s handbook of systematic reviews for interventions [15].

Literature Search

PubMed, Scopus, Web of Science, Virtual Health Library (VHL), Google Scholar, and Cochrane databases were searched using the following strategy “("Psoriasis" OR "psoriatic") AND [("Risankizumab " OR "BI 655066") OR ("Infliximab")]”. An online search was performed on clinicaltrials.gov to ensure adequate inclusion of all studies.

Eligibility Criteria

Included studies were selected according to the following criteria: (1) study design: randomized clinical trials. (2) population: adult patients with moderate-to-severe plaque psoriasis, (3) interventions: risankizumab 150mg or Infliximab 5mg, (4) comparator: placebo, (5) outcomes: psoriasis area-and-severity index (PASI) which includes PASI 75, PASI 90; static physician’s global assessment static physician’s global assessment (sPGA) clear; incidence of infection; incidence of any adverse side effect (AE); and incidence of serious adverse side effects (SAEs). Exclusion criteria were: (1) observational, retrospective studies, and non-controlled trials, (2) animal studies, in vitro studies, review articles, case reports, conference abstracts, and duplicate publications. (3) studies that are in a non-English language. (4) Studies whose full-text article was not available.

Studies Selection

Two independent reviewers performed title and abstract screening for matching studies. Then, full-text papers of included titles and abstracts were retrieved and screened independently. Disagreements were settled through discussion with a third reviewer.

Data Extraction

After obtaining full-text papers, two reviewers independently performed data extraction. Baseline data including sample size, drug administered, baseline psoriasis scores, body surface area involved, body mass index, white race, and previous treatment was extracted. A third reviewer resolved any disagreement between the two reviewers.

Data Synthesis and Analysis

Review Manager Software, Version 5.3 was utilized to perform data analysis. The risk ratios (RR) with the respective 95% confidence intervals (CIs) of different psoriasis scores were pooled in a meta-analysis model, using the Mantel-Haenszel method. Homogeneous data was analyzed under the fixed-effects model and used the random-effects model for heterogeneous data. Heterogeneity was resolved using Cochrane’s leave-one-out method [15]. 

This meta-analysis conducted a subgroup analysis according to the drug administered (risankizumab or infliximab) against placebo and tested for subgroup differences using chi-square test with its P-value. Heterogeneity among studies was assessed using I2 test and P-value from the chi-squared test of heterogeneity. Values of I2 >50 and P<0.1 are significant markers of heterogeneity among studies according to Cochrane’s handbook.

Risk of Bias Assessment

Risk of bias assessment was performed using Cochrane’s risk of bias tool [16]. It includes the following domains: sequence generation (selection bias), allocation sequence concealment (selection bias), blinding of participants and personnel (performance bias), blinding of outcome assessment (detection bias), incomplete outcome data (attrition bias), selective outcome reporting (reporting bias) and other potential sources of bias. The reviewers’ judgment is categorized as ‘Low risk’, ‘High risk’ or ‘Unclear risk’ of bias according to each domain. To assess the risk of bias within the included studies, two independent reviewers used the risk of bias assessment tool using Cochrane risk of bias tool [16]. The reviewers’ judgment is categorized as ‘Low risk’, ‘High risk’ or ‘Unclear risk’ of bias according to each domain.

Publication Bias

For the assessment of publication bias, the pooled effect estimate was plotted against its standered error (SE) in a funnel plot generated by RevMan software. Due to the small number of included studies (less than 10), funnel-plot-based methods could not be utilized.

Results

Results of Literature Search

**Figure 1:**
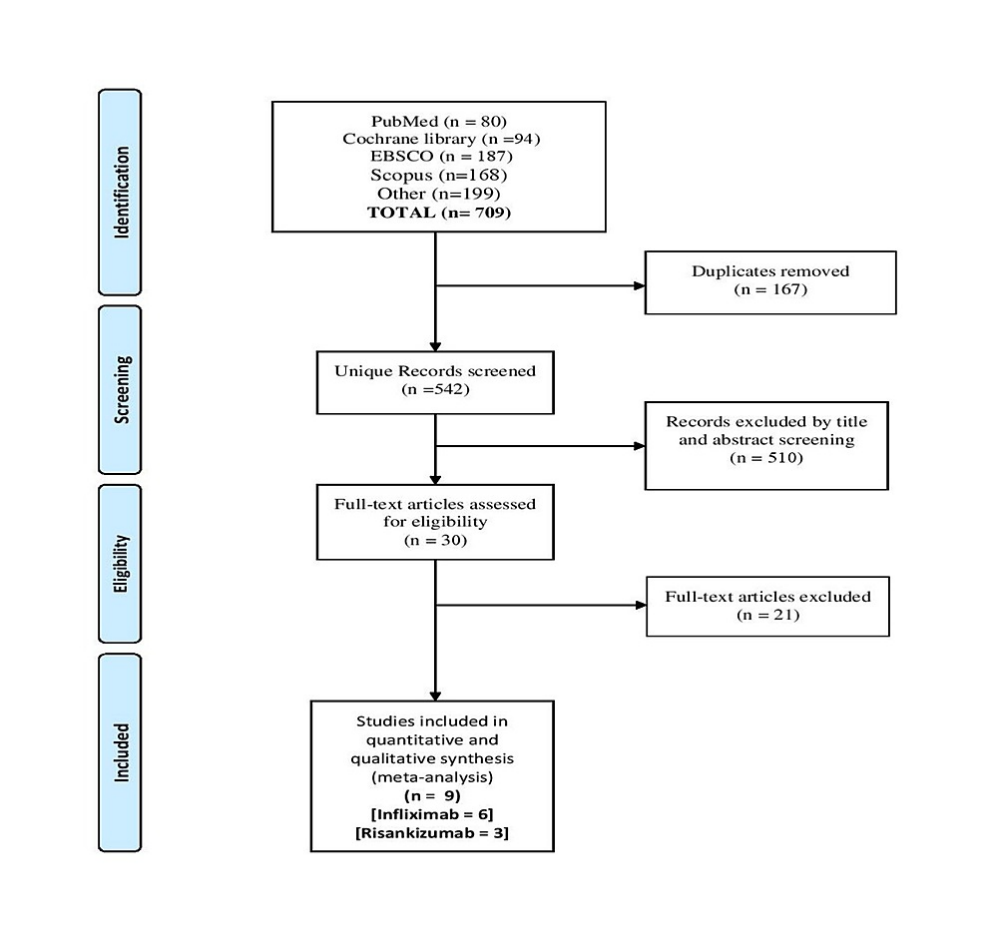
PRISMA flow diagram of the literature search PRISMA: Preferred Reporting Items for Systematic Reviews and Meta-Analyses

Searching databases yielded a total of 709 studies. After removing duplicates, 542 studies remained for the title and abstract screening. Excluded studies compromised of 510 studies as they did not meet the inclusion criteria. Nine studies were finally included after the full-text screening. The reviewers excluded 30 full-text papers, 17 of which were animal trials, eight reviews, and six trials compared risankizumab or Infliximab with other drugs (no placebo group). Figure 1 shows a PRISMA diagram for this study.

Results of Risk of Bias Assessment

**Figure 2:**
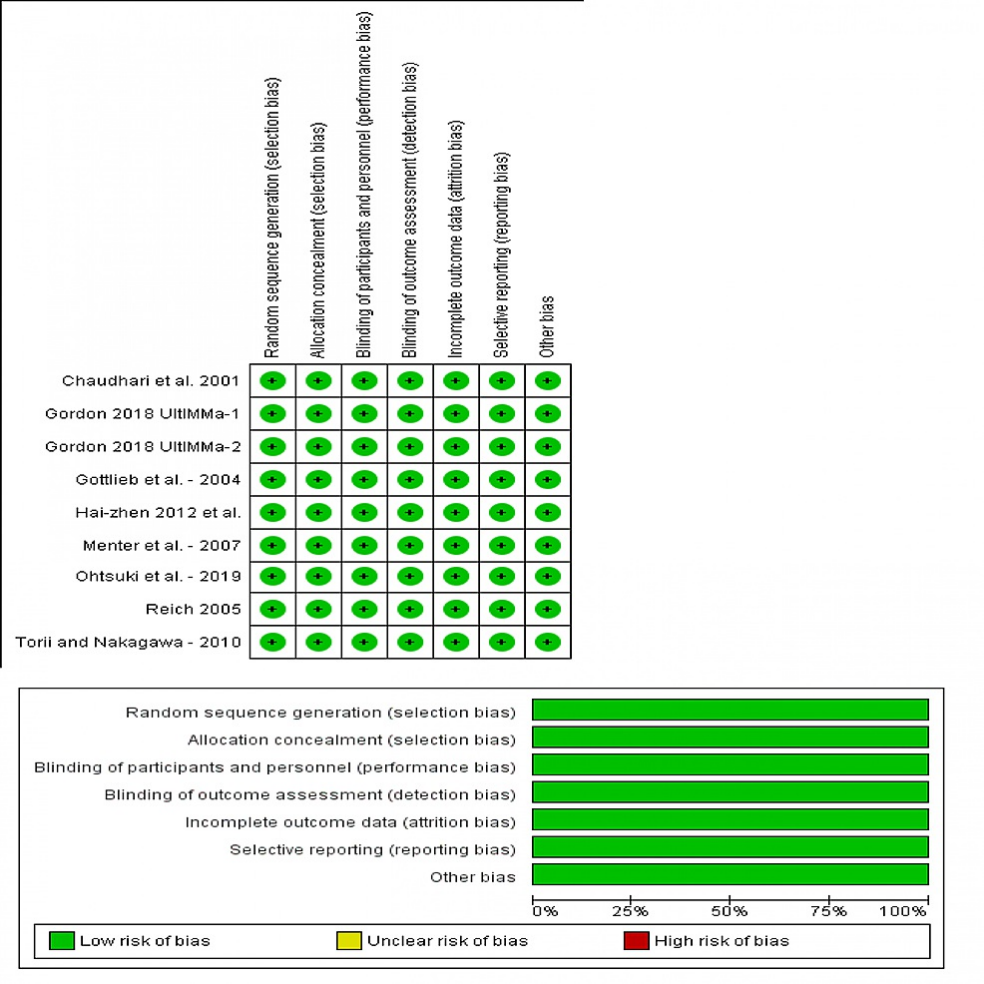
Risk of bias summary and graph of included trials Source: [17-22, 24-25]

This meta-analysis found an overall low risk of bias among the included studies. All studies adequately reported randomization, allocation concealment; they were all double-blinded, with no missing data or attrition bias. Figure 2 shows a summary and graph for the risk of bias of included studies. All studies were with low risk of bias.

Summary of Interventions

This meta-analysis included nine studies, six of which used infliximab [17-22] and three [23-25] used risankizumab. One trial included two steps, and were treated each step as a separate study [24]. A total of 1255 patients were enrolled in infliximab trials, compared with 1418 patients in the risankizumab group. Table 1 shows a summary for baseline characteristics of included trials.

**Table 1:** Summary for baseline characteristics of included trials

Study ID	Drug Administered	Study Design	Females, n(%)		Sample Size		Age, year, mean (SD)				Baseline PASI, mean (SD)		BSA involved, %, mean (SD)		White race, n(%)			Disease duration, years, mean (SD)	
			Drug	Placebo	Drug	Placebo	Drug	Placebo	Drug			Placebo	Drug	Placebo	Drug		Placebo	Drug	Placebo
Chaudhari et al. [17]	Infliximab	RCT	4 (36%)	3 (27%)	11	11	51 (14)	45 (12)	26.6 (10.3)			20.3 (5.5)	N/A	N/A	N/A		N/A	N/A	N/A
Gottlieb et al. [18]	Infliximab	RCT	26 (26%)	20 (39%)	99	51	44 (9.5)	45 (8.87)	20 (5.8)			18 (3.56)	25 (2.33)	26 (4.1)	N/A		N/A	16 (3.8)	16 (8.1)
Yang et al. [19]	Infliximab	RCT	24 (28.5%)	10 (22%)	84	45	39.4(12.3)	40.1(11.1)	N/A			N/A	N/A	N/A	N/A		N/A	16 (10.8)	16 (8.9)
Menter et al. [20]	Infliximab	RCT	110 (35%)	62 (29.8%)	314	208	44.5 (13)	44.4 (12.5)	20.4 (7.5)			19.8 (7.7)	28.7(16.4)	28.4 (17.6)	293 (93%)		189 (91%)	19.1 (11.7)	17.8 (10.8)
Reich et al. [21]	Infliximab	RCT	94 (31%)	16 (21%)	301	77	42.6 (11.7)	43.8 (12.6)	22.9 (9.3)			22.8 (8.7)	34.1 (19)	33.5 (18)	N/A		N/A	19.1 (11.0)	17.3 (11.1)
Torii et al. [22]	Infliximab	RCT	13 (37%)	5 (26.3%)	35	19	46.9 (13)	43.3 (12.3)	N/A			N/A	N/A	N/A	N/A		N/A	14.5(8.9)	11.1(6.5)
Gordon et al. [24]	Risankizumab	RCT	92 (30%)	23(23%)	304	102	48·3 (13·4)	49·3 (13·6)	20·6 (7·7)			20·5 (6·7)	26·2% (15·4)	27·9% (17·2)	200 (66%)		71 (70%)	N/A	N/A
Gordon et al. [24]	Risankizumab	RCT	91 (31%)	31 (32%)	294	98	46·2 (13·7)	46·3 (13·3)	20·5 (7·8)			18·9 (7·3)	26·2% (15·9)	23·9% (15·7)	255 (87%)		87 (89%)	N/A	N/A
Blauvelt et al. [23]	Risankizumab	RCT	124 (30.4%)	27 (27%)	407	100	49.6 (13.17)	47.9 (13.78)		N/A		N/A	N/A	N/A	320 (78.6%)		82(82%)	N/A	N/A
Ohtsuki et al. [25]	Risankizumab	RCT	10 (17.2%)	13 (22.4%)	55	58	53.3 (11.9)	50.9 (11.2)	26.3 (11.7)			24.0 (9.1)	40.5 (22.7)	33.2 (19.0)		N/A	N/A	N/A	N/A
RCT: Randoimzed clinical trials, PASI: Psoriasis Area & Severity Index, BSA: Body surface area Data are represented as n(%) or mean(SD) unless stated otherwise																			

Efficacy Outcomes

**Figure 3:**
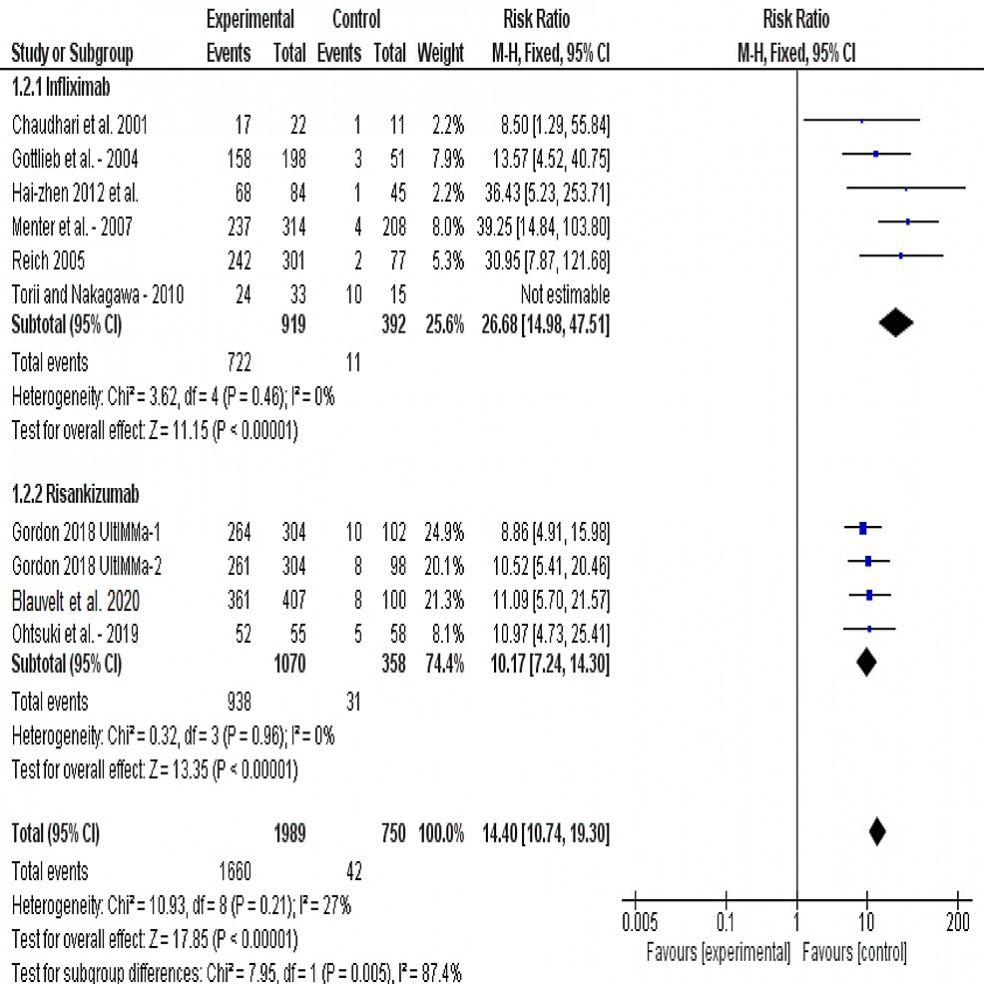
Forest plot of analysis of PASI-75 outcome Source: [17-22, 24-25]. PASI: Psoriasis area-and-severity index

PASI-75: All studies reported PASI-75 score. The overall risk ratio of both drugs showed a significantly high therapeutic effect (RR= 14.40, 95% CI [10.74, 19.30], p<-0.001). Pooled analysis was homogeneous (I2=27%, P=0.2). This meta-analysis found that both infliximab and risankizumab significantly (p<0.001) increase the number of patients with 75% improvement on the PASI scale, (RR= 26.68, 95% CI [14.98, 47.51]) and (RR= 10.17, 95% CI [7.24, 14.30]) respectively. Test for subgroup differences showed that risankizumab is significantly superior to infliximab in increasing the number of improved patients >75% on PASI Score (p=0.005) (Figure 3).

**Figure 4:**
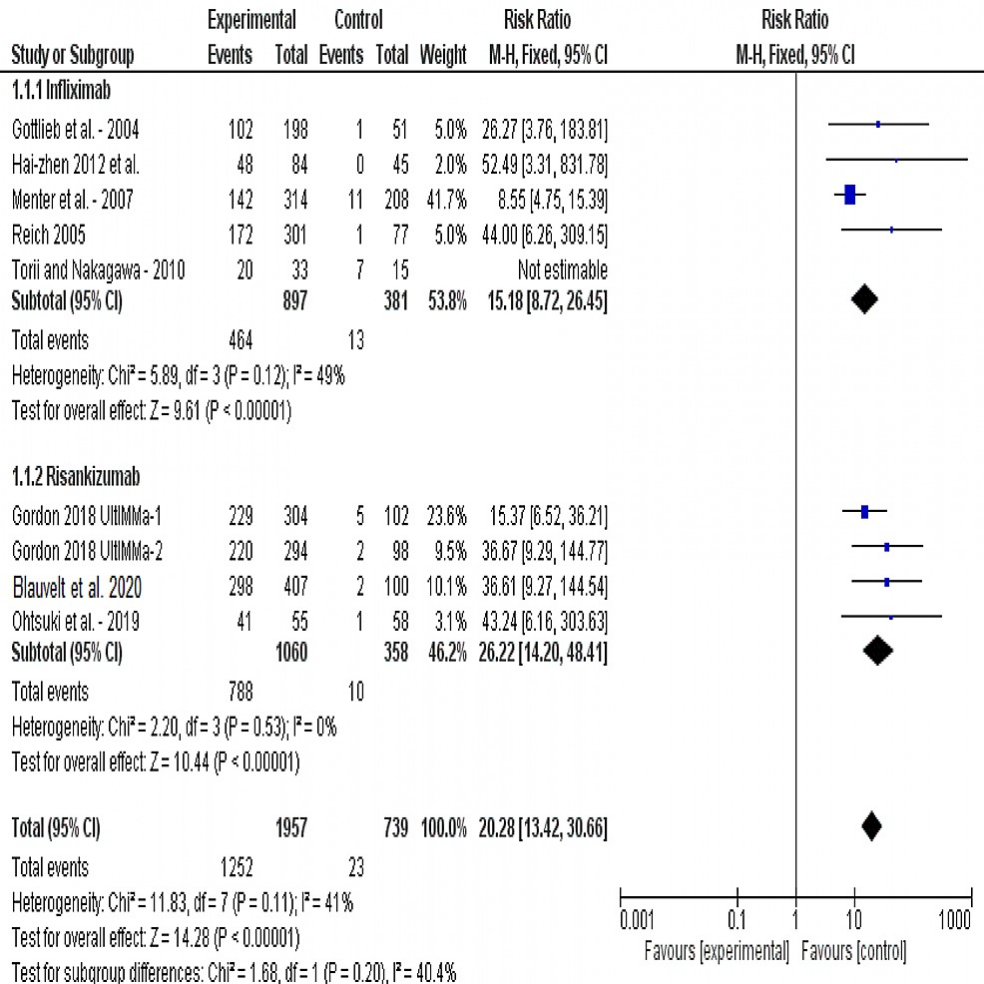
Forest plot of analysis of PASI-90 outcome Source: [17-22, 24-25], PASI: Psoriasis area-and-severity index

PASI-90: The combined risk ratio of interventions showed a significant high therapeutic effect (RR= 20.28, 95% CI [13.42, 30.66], p<-0.001) as reported by eight studies. Pooled analysis was homogeneous (I2=41%, P=0.1). Risankizumab has shown to be effective than placebo (RR= 26.22, 95% CI [14.20, 48.41], p<0.001), and infliximab revealed similar results (RR= 15.18, 95% CI [8.72, 26.45], p<0.001). Test for subgroup differences showed no statistically significant difference between the two interventions (p=0.2) (Figure 4).

**Figure 5:**
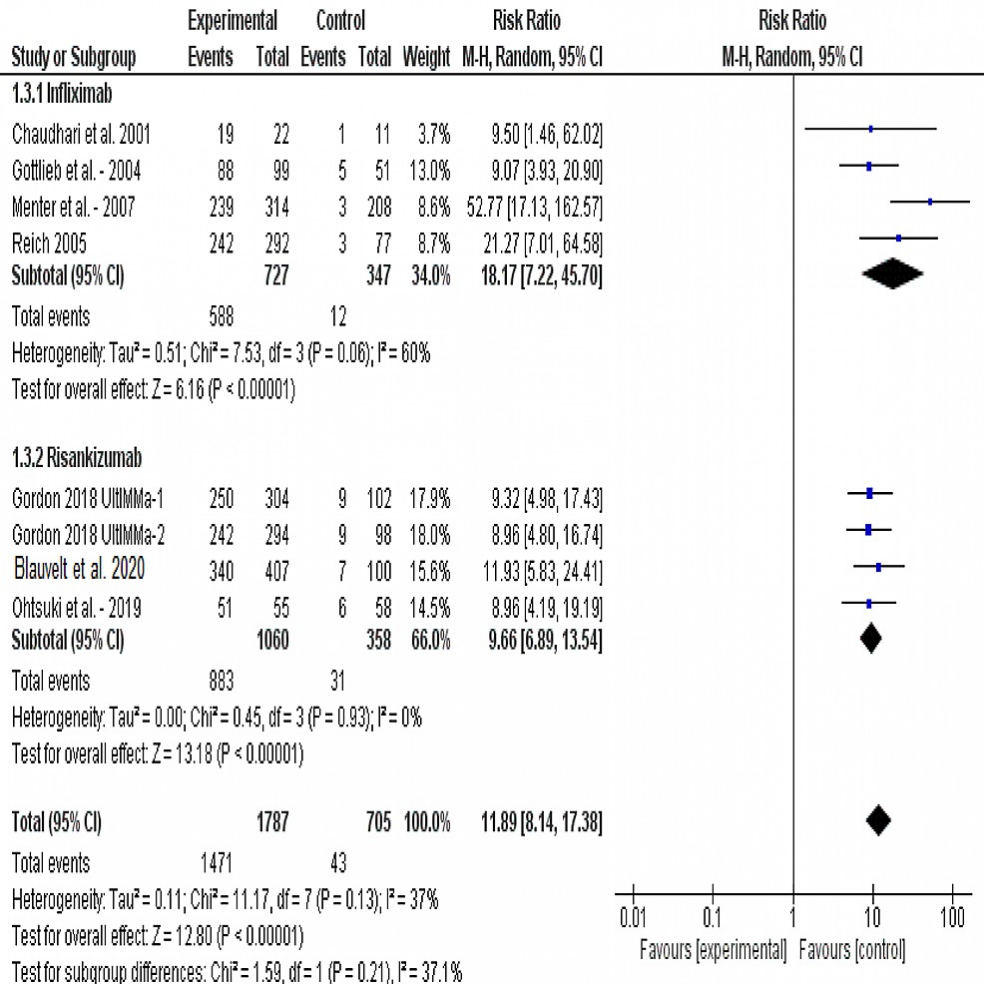
Forest plot of analysis of sPGA outcome Source: [17-22, 24-25]; sPGA: Static physician’s global assessment

sPGA at week 10: All studies (except Tori et al.) reported sPGA score improvements at week 10. The combined risk ratios of both drugs favored the experimental group (RR= 11.89, 95% CI [8.14, 17.38], p<0.001). Infliximab was found to be associated with significant sPGA improvements compared to placebo (RR= 18.17, 95% CI [7.22, 45.70], p<0.001). Similar results were found for risankizumab as well, (RR= 9.66, 95% CI [6.89, 13.54], p<0.001). Test for subgroup differences did not reveal any significant subgroup effect (p=0.21). Pooled results were homogeneous (I2=37%, P=0.13) (Figure 5).

Safety Outcomes

**Figure 6:**
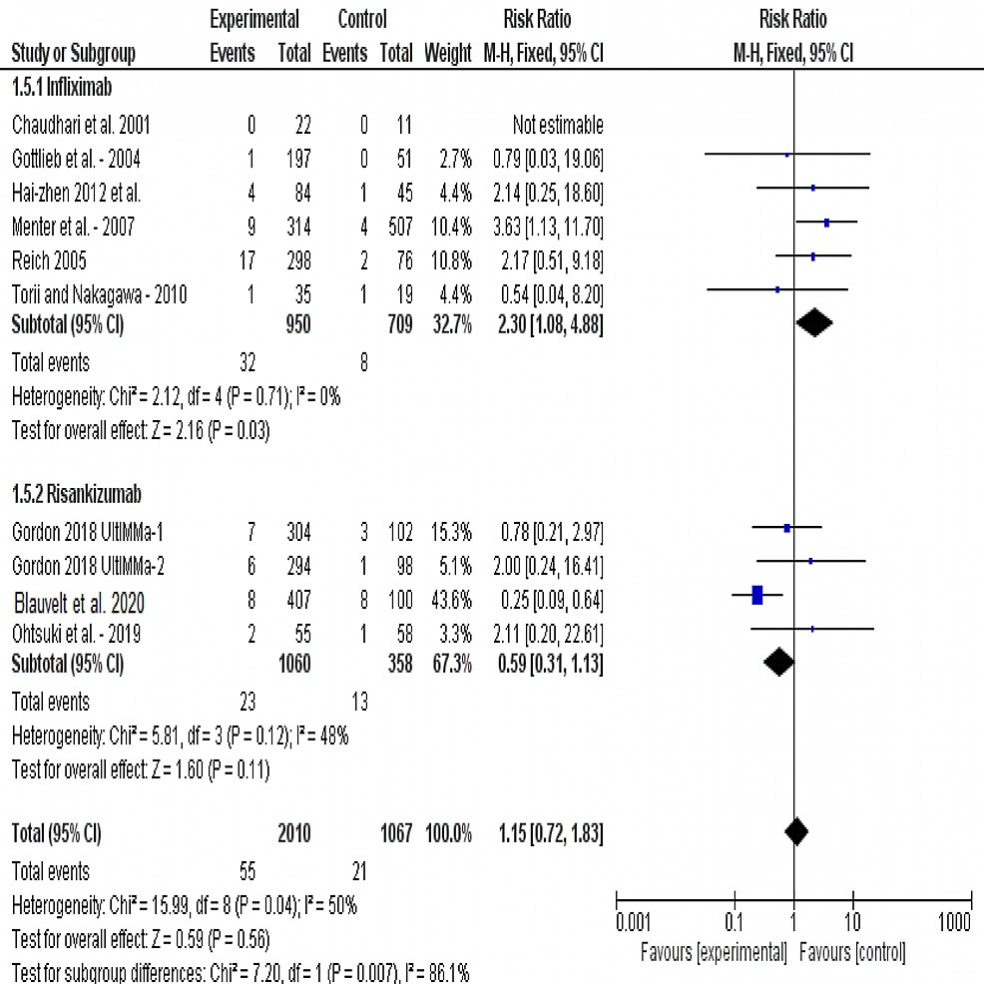
Forest plot of analysis of serious adverse events outcome Source: [17-22, 24-25]

Incidence of serious adverse events (SAEs): The overall risk ratio of interventions, as reported by all studies, showed no significant occurrence of side effects (p=0.56). Pooled results were homogenous (I2=50%, P=0.04). Compared to placebo, risankizumab does not cause significant serious side effects (RR = 0.59, 95% CI [0.31, 1.13], p=0.12). While on the other hand, infliximab causes significant serious adverse events (RR = 2.30, 95% CI [1.08, 4.88], p=0.03). Test for subgroup difference showed that risankizumab is much safer than infliximab (p<0.001), Figure 6.

**Figure 7:**
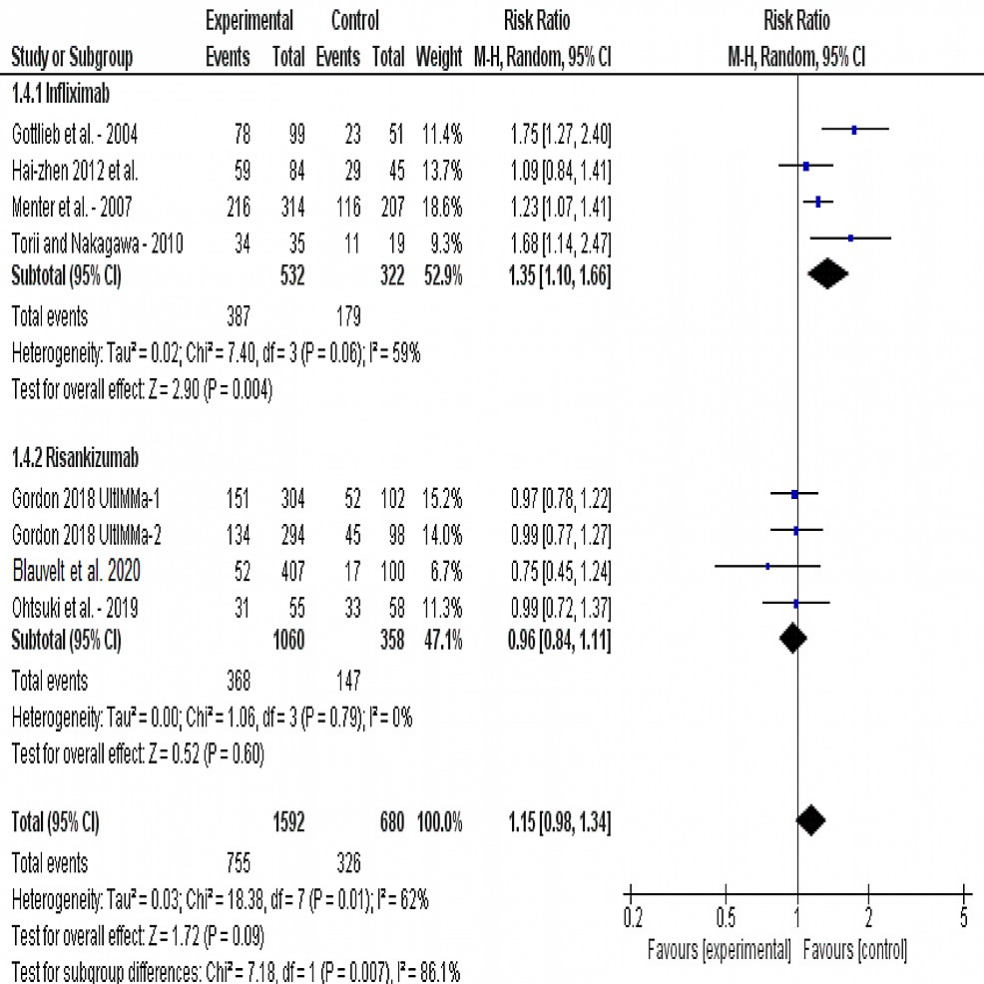
Forest plot of analysis of the incidence of any adverse events outcome Source: [17-22, 24-25]

Incidence of any adverse event (AE): The incidence of any adverse event was reported by all studies. This meta-analysis found that infliximab and risankizumab are highly associated with an increased incidence of adverse events compared to placebo (RR= 1.15, 95% CI [0.98, 1.34], p=0.01). The overall risk ratio showed that (1) infliximab is significantly associated with more adverse events than placebo (RR= 1.35, 95% CI [1.10, 1.66], p<0.001), and (2) risankizumab does not cause significant side effects when compared to placebo (RR= 0.96, 95% CI [0.84, 1.11], p=0.51) (Figure 7). Test for subgroup differences regarding the incidence of any adverse events highly favored risankizumab (p=0.007). Pooled analysis was heterogeneous (I2 = 62%, P=0.01). Heterogeneity was best resolved by excluding Gottlieb et al. [18] Homogeneous results showed that risankizumab is still significantly safer than infliximab (p=0.03).

**Figure 8:**
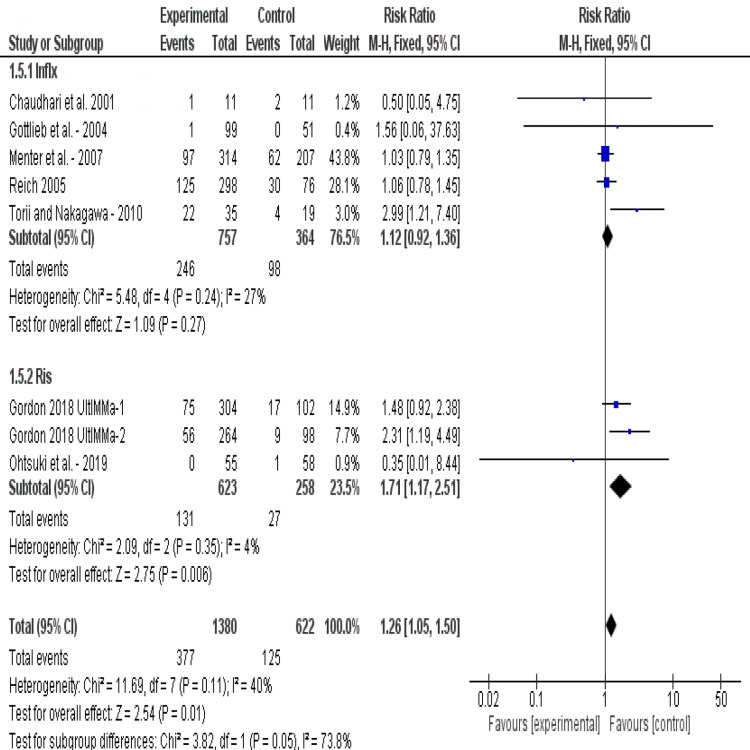
Forest plot of analysis of the incidence of infection outcome Source: [17-22, 24-25]

Incidence of Infection: The overall risk ratios show that infection is significantly evident in the treatment group (RR=1.26, 95% CI [1.05, 1.50], p=0.01). Subgroup analysis shows that infliximab does not cause significant infections compared to placebo (RR=1.12, 95% CI [0.92, 1.36], p=0.27). Risankizumab is associated with significant incidence of infection (RR = 1.71, 95% CI [1.17, 2.51], p=0.006). Test for subgroup differences shows that there is no significant difference between both drugs in terms of infection (p=0.05). Pooled results were homogeneous (P=0.11, I2=40%) (Figure 8).

Discussion

The results of this meta-analysis provide a class one evidence that risankizumab is significantly superior to infliximab for the treatment of plaque psoriasis in both efficacy and safety. This meta-analysis found that, when indirectly comparing both drugs, risankizumab leads to more improved patients > 75% on the PASI score. Regarding PASI-90 and sPGA outcomes, both drugs exerted the same efficacy. As for safety endpoints, subgroup difference tests show that infliximab is significantly associated with more incidence of any adverse events and serious adverse events. Test for subgroup differences showed no significant difference between both drugs in terms of infection.

Although four of included studies used risankizumab compared to six studies used infliximab, the risankizumab trials included a larger sample size than infliximab group. The literature is full of secondary works that compare risankizumab with other drugs. A recent network meta-analysis [8] compared risankizumab with brodalumab, secukinumab, ixekizumab, ustekinumab, guselkumab, and tildrakizumab. The results showed that risankizumab has the highest efficacy and lowest risks. Risankizumab 150mg has shown to improve PASI 100 scores in Japanese patients [26]. Regarding the safety profile, the drug has shown to be safe and not associated with significant side effects [27].

Another meta-analysis including 13 studies of IL-inhibitors trials found that risankizumab was better tolerated than other drugs [28]. Another recent systematic review and network meta-analysis performed including trials using IL-inhibitors found the same results [29]. A systematic review conducted last year found similar results as well .

A systematic review found that infliximab is significantly associated with relief of psoriatic symptoms and skin improvements. The study also found that infliximab causes significant pain and infusion reactions [30]. Moreover, another study found that in patients with inflammatory bowel disease, infliximab led to relief of psoriatic symptoms in 87% of participants, and that resolution occurred in 48% of cases as a result of resolution of the drug [30]. A recent network meta-analysis found that infliximab has the highest efficacy compared with other anti-TNF drugs [12]. The results from previous network meta-analysis suggests that infliximab is associated with significant side effects.

In this meta-analysis, an indirect comparison of risankizumab and infliximab provided a preliminary solution to the debate about which class is better; anti-TNFs or IL-23 inhibitors. This gives this study a point of strength. This meta-analysis included only randomized, placebo-controlled, clinical trials to provide strong evidence. All the studies were recent studies, published after the year 2000. This is evidenced by the fact that the methodology of all included trials was adequately performed, and that all studies were at low risk of bias in all domains. A total of 2673 patients were enrolled; this large sample size is also a point of strength to the evidence this paper provides.

However, the absence of clinical trials that directly compare risankizumab and infliximab is the main limitation. This study's findings are based on tests of subgroup differences. Although this meta-analysis is supported by all previous meta-analyses, a direct comparison is necessary to show the “true” findings of both drugs in terms of efficacy and safety. Another limitation is that this meta-analysis could not enter some outcomes into analysis such as dermatology life and quality index, due to the dichotomization of the outcome in some studies. Future head to head clinical trials to compare both drugs in terms of efficacy and safety outcomes is recommended.

## Conclusions

Risankizumab is safer and is associated with significantly more improvements than infliximab. Future studies should provide a direct comparison between the risankizumab and infliximab in term of safety and efficacy.
